# The complete chloroplast genome sequence of the medicinal and economic plant woad *Isatis indigotica* (Brassicaceae)

**DOI:** 10.1080/23802359.2017.1361356

**Published:** 2017-08-08

**Authors:** Shu Yang, Zhezhi Wang

**Affiliations:** Key Laboratory of the Ministry of Education for Medicinal Resources and Natural Pharmaceutical Chemistry, National Engineering Laboratory for Resource Development of Endangered Crude Drugs in Northwest China, College of Life Sciences, Shaanxi Normal University, Xi’an, Shaanxi, P.R. China

**Keywords:** *Isatis indigotica*, Illumina sequencing, chloroplast genome, MITObim

## Abstract

Chloroplast (cp) genome sequences provide a valuable source for phylogenetic analysis. It becomes a popular useful tool for population and phylogeny in recently report. Here, the complete chloroplast genome of the *Isatis indigotica* has been reconstructed from the whole-genome Illumina sequencing data. The circular genome is 156,670 bp in size and comprises a pair of inverted repeat (IR) regions of 26,995 bp each, a large single-copy (LSC) region of 84,907 bp and a small single-copy (SSC) region of 17,773 bp. The total GC content is 36.5%, while the corresponding values of the LSC, SSC and IR region are 34.2%, 29.7% and 42.3%, respectively. The chloroplast genome contains 140 genes, including 94 protein-coding genes. The phylogenetic analysis showed a strong sister relationship with *Raphanus sativus* in Brassicaceae. These findings provide a foundation for further investigation of cp genome evolution in *I. indigotica* and other higher plants.

The chloroplast is a plant-specific organelle that carries out photosynthesis and other crucial biosynthesis, which becoming valuable phylogenetic tool for low recombination rate (Saski et al. 2007; Dong et al. 2012). It is hot spot that researches on sequencing chloroplast of valued medicine plants such as *Salvia miltiorrhiza*, *Magnolia officinalis* and *Ginkgo biloba* (Li et al. 2012; Lin et al. 2012; Qian et al. 2013).

*Isatis indigotica* Fort. (Brassicaceae) is native to the steppe and desert zones of the Caucasus as a source of blue dye. In China, *I. indigotica* is widely distributed and used as a popular herbal medicine for treatment of colds, fever and influenza (Xu et al. 2010; Shan et al. 2014). The gene resource of *I. indigotica* is abundant (Tang et al. 2014; Zhang et al. 2016); however, few data are available regarding the *I. indigotica* chloroplast genome. Here, we collected the samples of *I. indigotica* from Qinling Mountains (Shaanxi Province) and stored in our lab and then reconstructed the complete chloroplast genome sequence from the whole-genome Illumina sequencing data. Our data are valuable for future genetic and phylogenetic studies on *I. indigotica*.

A subset of 17.4 M trimmed reads were used for reconstructing the chloroplast genome with MITObim v1.7 (Hahn et al. 2013), with that of its congener *Arabidopsis thaliana* (GenBank: NC_000932) as the initial reference sequence. A total of 1,524,092 individual chloroplast reads were achieved with an average coverage of 1223.6-fold. The chloroplast genome was annotated in GENEIOUS R8 (Biomatters Ltd., Auckland, New Zealand) by aligning with that of *A. thaliana* (NC_000932).

The whole chloroplast genome of *I. indigotica* is a double-stranded circular DNA molecule with a length of 156,670 bp with the accession number KT939360. It shows a typical quadripartite structure which comprises a pair of inverted repeat (IR) regions of 26,995 bp each, separated by a large single-copy (LSC) region of 84,907 bp and a small single-copy (SSC) region of 17,773 bp. The total GC content is 36.5%, while the corresponding values of the LSC, SSC and IR region are 34.2%, 29.7% and 42.3%, respectively.

This chloroplast genome harbours 137 functional genes, including 90 protein-coding genes (PCGs), 37 tRNA genes and 8 rRNA genes. Eight PCGs, seven tRNA genes and all rRNA genes, most of which related to photosynthesis, are duplicated in the IR regions. The LSC region possesses 61 PCGs and 22 tRNA genes, while the SSC region contains 13 PCGs and one tRNA gene. 32 PCGs, 19 tRNA genes and 4 rRNA genes are located in the forward strand while others are located in the reverse strand.

We performed a phylogenetic tree using 16 cp genome sequences in Brassicaceae family. The neighbour-joining tree showed the position of *I. indigotica* was situated as the sister of *Raphanus sativus* which was in good agreement with taxonomical classification (Figure 1). This study provides the reference to phylogenetic and evolutional study.

**Figure 1.**
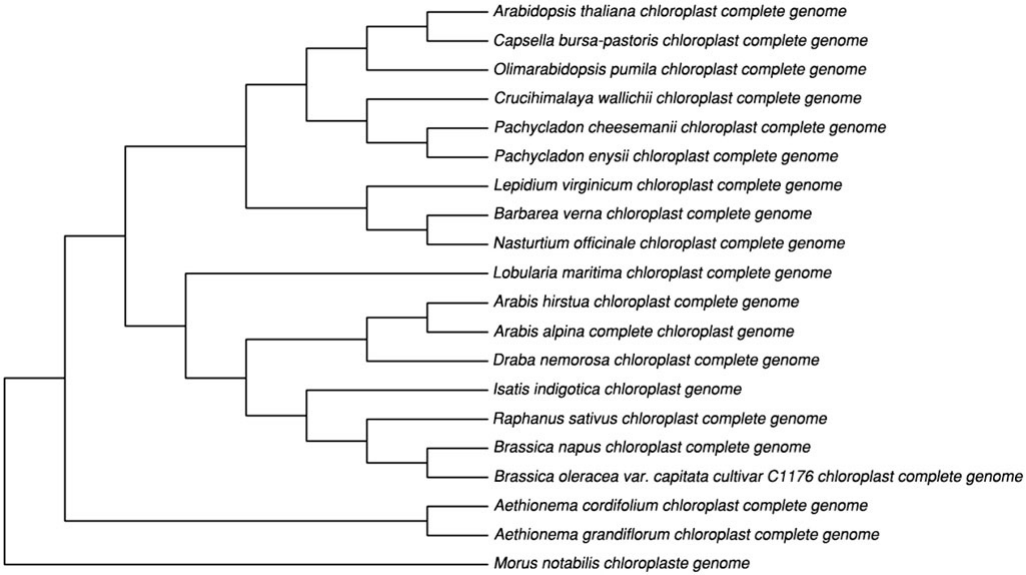
Phylogenetic of 16 species within the family Brassicaceae based on the neighbour-joining analysis of the whole cp genome sequences using 500 bootstrap replicates and setting *Morus notabilis* (Moraceae) as out-group.
